# PFGE diversity within the methicillin-resistant *Staphylococcus aureus *clonal lineage ST398

**DOI:** 10.1186/1471-2180-10-40

**Published:** 2010-02-09

**Authors:** Thijs Bosch, Albert J de Neeling, Leo M Schouls, Kim W van der Zwaluw, Jan AJW Kluytmans, Hajo Grundmann, Xander W Huijsdens

**Affiliations:** 1 Centre for infectious disease control, National institute for public health and the environment (RIVM), Bilthoven, the Netherlands; 2 Department of medical microbiology and infection control, VU medical centre, Amsterdam, the Netherlands; 3 Laboratory for microbiology and infection control, Amphia hospital, Breda, the Netherlands

## Abstract

**Background:**

Livestock has recently been identified as a new reservoir of methicillin-resistant *Staphylococcus aureus *(MRSA). Most isolates belong to ST398 and are non-typeable with PFGE using *Sma*I, making it difficult to study transmission and outbreaks. Therefore, a new PFGE using *Cfr*9I, a neoschizomer of *Sma*I was optimized and evaluated to investigate ST398 isolates.

**Results:**

After optimizing and evaluating the *Cfr*9I PFGE, clear and reproducible banding patterns were obtained from all previously non-typeable MRSA (NT_*Sma*I _-MRSA) isolates. The PFGE patterns of ST398 isolates showed more diversity than with *spa*-typing and/or MLST. The PFGE results showed diversity within and between the two most prevalent *spa*-types of NT_*Sma*I _-MRSA (t011 and t108). No match was found, when comparing banding patterns of the NT_*Sma*I _-MRSA with 700 different PFGE types, obtained with *Sma*I digestion, in our database of more than 4000 strains. Furthermore, possible transmission among veterinarians and their family members was investigated and an outbreak of ST398 MRSA in a residential care facility was confirmed with the *Cfr*9I PFGE.

**Conclusions:**

The adjusted PFGE can be used as a method for selecting important and distinct ST398 isolates for further research. The adjustments in the PFGE protocol using *Cfr*9I are easy to implement to study the ST398 clonal lineage in laboratories which already have a PFGE facility.

## Background

Methicillin-resistant *Staphylococcus aureus *(MRSA) is a major cause of nosocomial and community-associated infections worldwide. Most cases of community-associated MRSA (CA-MRSA) have been associated with skin and soft-tissue infections in previously healthy individuals [1,2]. Since 2003, pigs [3-7] and other animals such as horses [8,9], poultry [10] and calves [11] have been identified as a new reservoir for CA-MRSA. Most of the livestock related MRSA strains share the same multi locus sequence typing (MLST) type, namely ST398. Throughout Europe [9,12-14], Canada [6] and in the United States [15] ST398 has been found in association with animal husbandry, indicating a worldwide clonal lineage. Although the clinical importance of ST398 is still controversial, there are reports indicating transmission and infections among humans [16-18]. Pulsed Field Gel Electrophoresis (PFGE) using *Sma*I is considered to be the gold standard for typing MRSA isolates [19]. When PFGE was performed on ST398 isolates, no banding patterns could be generated, due to methylation of the *Sma*I site [20]. Therefore, ST398 isolates are referred to as PFGE non-typeable (NT_*Sma*I_)-MRSA. Some years ago staphylococcal protein A (*spa*) typing was introduced as a highly discriminatory typing method to characterize *S. aureus *isolates [21,22]. However, *spa*-typing of the ST398 isolates revealed very limited variation within this group and 80% of our ST398 isolates had either *spa*-type t011, t108 or t034 [23]. Recently, a multiple-locus variable number of tandem repeat analysis (MLVA) has been presented [24]. Although MLVA is significantly more discriminatory than *spa*-typing, it was unable to yield a better discrimination of the isolates of the ST398 lineage. The lack of a typing method that can discriminate ST398 strains has hampered studies on the origin and transmission routes of this MRSA clade.

In the Netherlands all first MRSA isolates obtained from patients with staphylococcal disease and from patients that carry the pathogen are sent to the National MRSA reference centre for typing. In 2007, 30% of all forwarded MRSA isolates were NT_*Sma*I _-MRSA [23].

Recently, a neoschizomer of *Sma*I, designated as *Cfr9*I, was shown to be insensitive for the DNA-methylation leading to NT_*Sma*I _-MRSA isolates. In two studies this restriction enzyme was used for generating PFGE profiles of NT_*Sma*I _-MRSA isolates [18,25]. In the study presented here we optimized PFGE with restriction enzyme *Cfr*9I and evaluated its use to characterize NT_*Sma*I _-MRSA isolates.

The data will yield important information about the genetic diversity of the ST398 clonal lineage in the Netherlands and demonstrates that *Cfr*9I PFGE is a powerful tool to study possible transmission and outbreaks of MRSA isolates, previously not typeable by conventional PFGE approaches.

## Methods

### Bacterial isolates

The National Institute for Public Health and the Environment (RIVM) serves as the Dutch National MRSA reference center. All first MRSA isolates, one per patient, are sent to the RIVM for further typing. PFGE was carried out using restriction enzyme *Sma*I according to the Harmony protocol [26]. From this large MRSA collection a number of NT_*Sma*I _-MRSA was selected to optimize and validate the *Cfr*9I PFGE. To study the genetic diversity of the two most prevalent *spa*-types among NT_*Sma*I _-MRSA in the Netherlands, 60 NT_*Sma*I _-MRSA isolates (t011 (n = 30) and t108 (n = 30)) in 2008 from patients living in geographical dispersed regions in the Netherlands were used. In addition, 16 strains (8 pairs) from veterinarians and one of their family members, the latter whom did not have contact with animals and 40 pig and pig farmer isolates and 6 strains from an NT_*Sma*I _-MRSA outbreak in a residential care facility [18] were included in this study to assess the potential of the *Cfr*9I PFGE to identify transmissions. To validate the *Cfr*9I PFGE method, 10 typeable MRSA (T-MRSA) isolates and the reference strain NCTC 8325 were tested. Five non-typeable isolates were repeated 3 times with *Cfr*9I PFGE to ensure the reproducibility of the method.

### Molecular typing

All isolates were characterized with *spa *typing [22]. Spa-types were assigned using Bionumerics software version 5.1 (Applied Maths, Sint-Martens-Latem, Belgium). SCC*mec *typing of the isolates was performed using the multiplex PCR described by Boye et al [27].

In order to obtain clear and reproducible PFGE banding patterns using *Cfr*9I as restriction enzyme, the Harmony PFGE protocol had to be adjusted. This resulted in the following protocol: From each isolate, 100 μl bacterial suspension of an overnight Trypton Soy Broth (TSB) culture, was embedded in a plug mold (Biorad) with 1.2% low-melting-point agarose (Seakem gold^®^, Biorad). Then, 500 μl lysostaphine (100 μg/ml, Sigma) was added and incubated for 6 h at 37°C. Subsequently, the plugs were incubated overnight at 55°C with 500 μl Proteinase K (50 μg/ml, Merck). The plugs were then washed, 6 to 10 times in a shaking incubator for 30 min. in 1 × Tris-EDTA buffer (Fluka, pH 7) at 50°C in order to remove cell debris. Finally, the plugs were equilibrated in 1 × *Cfr*9I buffer (Fermentas, Ontario, Canada) for 15 min. at room temperature prior to digestion and then submerged in 200 μl of 1 × *Cfr*9I reaction buffer containing 40 U of *Cfr*9I restriction enzyme (Fermentas, Ontario, Canada). The reaction tubes were incubated overnight at 37°C in a shaking incubator. Further steps were carried out according to the Harmony protocol [26]. Briefly, a 1% agarose gel was poured into a gel tray and positioned in a contour-clamped homogeneous electric field (CHEF) (Biorad) tank and submerged in 1,700 ml of 0.5 × Tris-Borate-EDTA (TBE). The total run time was 22 h at 14°C with an initial pulse time of 5 s, a final pulse time of 50 s and a voltage of 6 V/cm or 200 V. Gels were stained in ethidium bromide (1 μg/ml, Invitrogen) and viewed and photographed with UV transillumination. Digital images were analyzed using Bionumerics software, version 5.1. If a difference in PFGE pattern was observed, a new pulsed field type was assigned. The definition of a PFGE cluster was based on a similarity cutoff of 80% [28] (Dice coefficient, represented by UPGMA, 0.5% optimization and 1.0% tolerance). Different PFGE clusters were given in alphabetical order. Every band difference within a PFGE cluster resulted in adding a numerical order to the pulsed field cluster.

## Results

### Optimization and validation of the *Cfr*9I PFGE method

In the initial experiments the *Sma*I restriction enzyme was replaced by *Cfr*9I and exactly the same conditions were used as in the original PFGE protocol. This led to uninformative PFGE patterns consisting mainly of smears and faint bands obtained through partial digestion of the genomic DNA. A higher lysostaphine concentration (100 μg/ml), longer incubation steps for lysis (6 h), proteinase K and digestion overnight and hot washes at 50°C - instead of washes at room temperature - produced clear and reproducible banding profiles.

After optimizing the PFGE method with *Cfr*9I, high quality banding patterns from all selected (n = 124) previously non-typeable ST398 MRSA isolates were obtained. For validation, both PFGE protocols (*Sma*I and *Cfr*9I) were performed on 10 typeable MRSA isolates and the reference strain NCTC 8325. Side-by-side comparison of *Sma*I and *Cfr*9I PFGE profiles yielded identical banding patterns consistent with unequivocal comparability of both restriction patterns. Reproducibility of the method was confirmed with 5 NT_*Sma*I _-MRSA isolates which were re-analyzed 3 times and yielded identical banding patterns.

#### Genetic diversity of NT_SmaI _-MRSA

All PFGE patterns of the NT_*Sma*I _-MRSA were compared with a database consisting of more than 4000 isolates containing over 700 different PFGE types obtained with *Sma*I digestion. Surprisingly, newly-obtained banding patterns of NT_*Sma*I _-MRSA isolates did not match with any known PFGE cluster in the national database of MRSA isolates collected since 2002.

**Figure 1:**
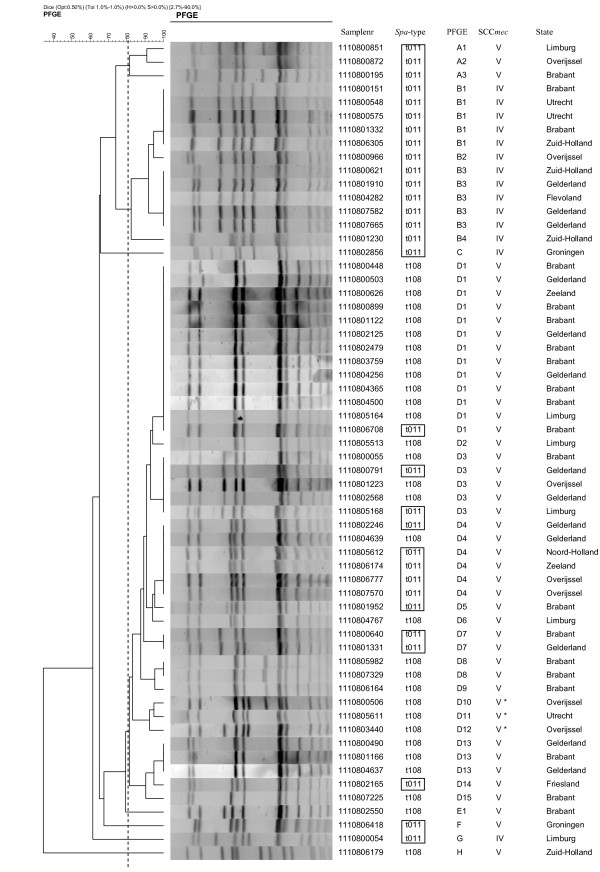
**Dendrogram of the *Cfr*9I PFGE results of NT_*Sma*I_-MRSA isolates with the 2 most prevalent *spa*-types in the Netherlands**.

Thirty t011 isolates revealed 16 different PFGE patterns (figure 1). The largest PFGE cluster consisted of 5 isolates, and 5 patterns were found more than once (n = 19). No correlation was found between PFGE cluster and geographic location. The minimal similarity (Dice coefficient, represented by UPGMA, 0.5% optimization and 1.0% tolerance) between the different patterns was 64% (data not shown). Thirty t108 isolates revealed 14 different PFGE patterns (figure 1). The largest cluster contained 12 isolates and 4 patterns were found more than once (n = 20). The clusters showed no geographical correlation. The minimal similarity of the t108 isolates was 50% (data not shown). One t108 isolate yielded a very distinct PFGE pattern (figure 1, pattern H). Without this isolate the minimal similarity of the t108 isolates would be 80%. The minimal similarity of the 60 NT_*Sma*I _-MRSA isolates was 35%, but most isolates share 80% or more similarity (figure 1). SCC*mec *typing of the 60 NT_*Sma*I _-MRSA isolates showed SCC*mec *type IV (n = 14) and SCC*mec *type V (n= 43). Three isolates yielded a variant of SCC*mec *type V (indicated in figure 1 with V*) and no SCC*mec *types I, II or III were found (figure 1).

#### Transmission of ST398 isolates

**Figure 2:**
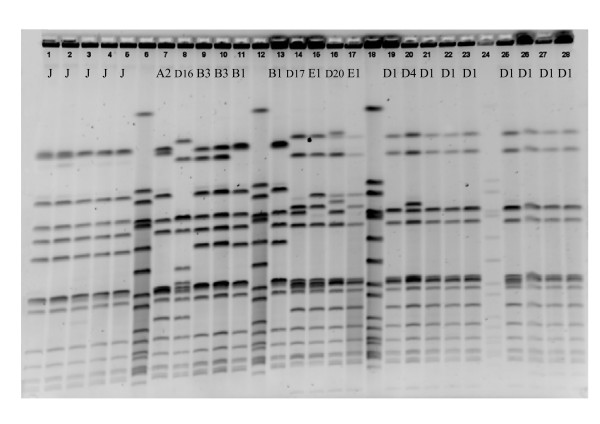
**PFGE patterns of ST398 isolates digested with *Cfr*9I restriction enzyme using NCTC 8325 as the reference standard**. Lanes 6, 12, 18, and 24, NCTC 8325; Lanes 1-5, isolates from an outbreak in a residential care facility, all PFGE pattern J; Lanes 7-8, and 14-15, two pairs of a veterinarian and a close family member with distinct PFGE patterns; Lanes 9-11, and 13, two pairs of a veterinarian and a close family member with identical banding patterns; Lanes 16-17, and 19-22, isolates of pig farm 6 with four different PFGE patterns; Lanes 23, and 25-28, isolates from pig farm 9 with identical banding patterns

The results of *Cfr*9I PFGE of 8 pairs of veterinarians and one of their close family members showed that 5 pairs gave indistinguishable banding patterns suggesting possible transmission of ST398 (figure 2 shows 2 pairs of indistinguishable banding patterns). Two pairs that did not match also had different *spa*-types (figure 2). One pair which had the same *spa-*type differed in a single PFGE band (data not shown). Six isolates belonging to an outbreak in a residential care facility with *spa*-types t2383 and t011 all shared the same banding pattern (figure 2). Furthermore, the transmission between pigs, pig farmers and their family on 9 different pig farms (table 1, figure 2) was studied. Farms 1 to 5 shared the same *spa*-type whereas on farms 6 to 9, two or more different *spa*-types were present. The number of different PFGE patterns (B1-K) differed between farms, ranging from indistinguishable patterns (farm 4) to 5 different PFGE patterns (farm 8). PFGE patterns B1, D1, D3, D4 and E1 were found on several farms (table 1). The minimal similarity within the farms varied from 52% (farm 5) to 100% (farm 4) and the minimal similarity between the farms was 61% (data not shown). Figure 2 shows the PFGE results of farm 6 with 4 different PFGE patterns and from farm 9 which all had indistinguishable PFGE patterns.

**Table 1:** Overview of transmission of ST398 MRSA on 9 farms (n = 40)

Strain nr	Farm	*spa*-type	Origin	PFGE pattern	Coefficient*
1110701181	1	t011	farmer	B3	70
1110700844	1	t011	pig	D7	

1110701184	2	t011	farmer	D4	86
1110700857	2	t011	pig	D4	
1110701182	2	t011	employee	E1	
1110701185	2	t011	relative	E1	

1110701429	3	t011	pig	B1	87
1110701595	3	t011	relative	B2	
1110701592	3	t011	farmer	D19	

1110701192	4	t108	farmer	D1	100
1110700908	4	t108	pig	D1	

1110701196	5	t567	farmer	D18	52
1110701197	5	t567	relative	D18	
1110700912	5	t567	pig	I	

1110701611	6	t108	dust	D1	84
1110701614	6	t108	dust	D1	
1110701604	6	t108	pig	D1	
1110701200	6	t011	farmer	D20	
1110701612	6	t011	dust	D4	
1110701605	6	t011	pig	D4	
1110701201	6	t011	relative	E1	

1110701600	7	t2741	employee	D14	95
1110701596	7	t011	farmer	D14	
1110701580	7	t011	pig	D14	
1110701601	7	t108	employee	D21	
1110701576	7	t011	pig	D21	
1110701577	7	t011	pig	D21	

1110700882	8	t011	pig	B1	66
1110700884	8	t108	pig	D1	
1110700876	8	t108	pig	D3	
1110700889	8	t2330	dust	D4	
1110701188	8	t2330	relative	D4	
1110701191	8	t2330	relative	D4	
1110700890	8	t108	dust	K	

1110701791	9	t108	dust	D1	86
1110701783	9	t108	pig	D1	
1110701788	9	t108	pig	D1	
1110703030	9	t108	relative	D1	
1110703031	9	t588	relative	D1	
1110703032	9	t108	relative	D3	

* Dice similarity coefficient, using UPGMA. Optimization 0,5%, position tolerance

## Discussion

MRSA isolates belonging to the ST398 clonal lineage are hard to discriminate based on *spa*-typing and/or MLST, hampering the assessment of transmission and outbreaks. Therefore, other techniques such as a modified PFGE could provide a new opportunity to differentiate ST398 isolates. The restriction enzyme *Sma*I does not cut the DNA of NT_*Sma*I _-MRSA isolates, due to methylation of the *Sma*I site. However, *Cfr*9I, a neoschizomer of *Sma*I, can be used for generating PFGE profiles of the NT_*Sma*I _-MRSA isolates. When the standard *Sma*I protocol was used for *Cfr*9I, banding patterns with smears and partial digests appeared. Other recently published articles seemed to have encountered similar problems with their *Cfr*9I PFGE [18,25]. The results indicated that lysis of ST398 isolates and digestion with restriction enzyme *Cfr*9I is more cumbersome than lysis of typeable MRSA and digestion with *Sma*I [29]. After modifying the protocol, banding patterns of similar quality as those of typeable MRSA isolates digested with *Sma*I were obtained. All previously non-typeable MRSA isolates can be typed with the optimized PFGE method providing a new opportunity to differentiate the ST398 clonal lineage.

From April 2002 until January 2008, all MRSA isolates sent to the RIVM have been typed with PFGE using *Sma*I as restriction enzyme creating a database with more than 4000 isolates with over 700 different PFGE types. Since *Cfr*9I recognizes the same restriction site as *Sma*I, *Cfr*9I enables analysis and comparison of the patterns with other profiles in our database. No comparison was found when comparing banding patterns of NT_*Sma*I _-MRSA with known PFGE patterns, suggesting that *Sma*I restriction modification is confined to a defined clonal lineage. Recently, ST398 isolates were typed using amplified fragment length polymorphism (AFLP). These data also suggested that ST398 is a distinct cluster recently introduced into the Dutch patient population [30].

The PFGE patterns of the two most prevalent *spa*-types (t011 and t108) within the NT_*Sma*I _-MRSA isolates showed more variation than *spa*-typing or MLST. The genetic diversity within the ST398 clonal lineage of MRSA sharing the same *spa*-type creates an opportunity for improved investigation of outbreak and potential transmission events. Spa-typing, which is currently used as a MRSA typing standard, cannot differentiate these isolates further. Using *Cfr*9I PFGE, *spa*-type t011 seemed to be more diverse than t108. Although the minimal similarity of the t108 isolates was 50%, this was mainly caused by a single isolate with a very distinct PFGE pattern (pattern H). Without this isolate the minimal similarity of the t108 isolates was 80%. The t011 isolates showed a minimal similarity of 64% (data not shown). SCC*mec *typing showed an almost equal distribution between SCC*mec *type IV (n = 14) and V (n = 16) for t011 isolates, whereas all t108 isolates carried SCC*mec *type V or a SCC*mec *type V variant. Huijsdens and colleagues performed SCC*mec *typing on 300 NT_*Sma*I _-MRSA isolates and they showed similar results [23]. This variation in SCC*mec *types may also indicates a higher diversity among t011 MRSA isolates compared to t108 isolates.

The minimal similarity of the *Cfr*9I PFGE patterns among ST398 isolates was 35% and showed variation within *spa*-types, but the diversity within this lineage is still limited. Furthermore, one isolate with *spa*-type t108 yielded a very distinct PFGE pattern which causes the similarity to be 35% (figure 1). When excluding this isolate from the dendrogram the minimal similarity was 62%. Comparing the PFGE results using the criteria by Tenover *et al*. and when a similarity cut-off of 80% was applied, most NT_*Sma*I _-MRSA isolates should be classified as one PFGE cluster [31,32]. However, the *Cfr*9I PFGE is still better in discriminating possible differences between NT_*Sma*I _-MRSA isolates.

No geographical relation could be found in either *spa*-type. However, most NT_*Sma*I _-MRSA isolates are found in areas with the highest pig density. This could be explained by the frequent movement of pigs between farms in the Netherlands. This facilitates the dissemination of ST398 MRSA on a national scale. A similar situation took place during the foot- and -mouth epidemic in England of 2001 [33].

To provide additional resolution on the molecular evolution and dissemination of MRSA lineages, several typing techniques such as PFGE, SCC*mec*- and *spa*-typing have been developed. Since PFGE with *Sma*I does not digest the DNA of ST398 isolates, *spa*-typing has been the method of choice for characterizing NT_*Sma*I _-MRSA isolates. However, given the low diversity in *spa*-types it is hard to ascertain health care-associated transmission if two or more different *spa*-types are present in the same institution. Fanoy *et al*. described an outbreak in a residential care facility where two *spa*-types (t2383 and t011) were prevalent [18]. After re-examination of the same isolates the PFGE profiles using *Cfr*9I were indistinguishable, indicating isogenicity. Moreover, the discriminatory ability of *spa*-typing of NT_*Sma*I _-MRSA is compromised by the fact that more than 80% of the NT_*Sma*I _-MRSA in the Netherlands belong either to *spa*-type t011 or t108 [23]. With the modified *Cfr*9I PFGE a better tool for epidemiological investigation has become available.

The results obtained by *Cfr*9I PFGE of isolates from veterinarians and their close family members showed possible transmission of ST398. Five out of eight pairs had identical profiles. The family members had themselves no contact with animals and were presumably infected by the occupationally exposed veterinarian. Two pairs of PFGE patterns among family members were not identical. Their isolates also had different *spa*-types. Family members may have been colonized by one MRSA through the veterinarian and subsequently the veterinarian may have been re-colonized by another MRSA after occupational exposure. One pair differed only in a single PFGE band probably as a consequence of micro-evolution.

A study on nine different farms revealed that the PFGE patterns of isolates from seven farms were related, but PFGE patterns varied within and between the farms. For example, farm 7, yielded only 2 very closely related PFGE patterns (D14, D21; similarity 95%), while other farms, like farm 8, showed 5 different PFGE patterns (B1, D1, D3, D4 and K) and had a similarity of only 66%. Different batches of animals entering the farm, carrying different NT_*Sma*I _-MRSA, could have caused variation within farms. Further study is needed to confirm that farms with a fast turnover of pigs indeed show a higher diversity of PFGE patterns of NT_*Sma*I _-MRSA.

## Conclusions

In conclusion, the modified PFGE protocol for *Cfr*9I provided highly informative banding patterns and showed good reproducibility. The PFGE results showed diversity within and between the two most prevalent *spa*-types among NT_*Sma*I _-MRSA. PFGE confirmed transmission of the ST398 clonal lineage within families and in a residential care facility. The modified PFGE approach can be used as a method for selecting important and distinct ST398 isolates for further research. The adjustments in the PFGE protocol using *Cfr*9I are easy to implement in laboratories which already have a PFGE facility, creating a powerful tool to study the ST398 clonal lineage.

## Authors' contributions

TB carried out all molecular typing and drafted the manuscript. AJN participated in the design of the study and revised the manuscript critically for important intellectual content. LMS has made substantial contributions to conception and design of the study. KWZ was responsible for analysis and interpretation of the data and revised the manuscript critically. JAJWK has been involved in drafting the manuscript and revising it critically for important intellectual content. HG participated in the design of the study and has given final approval of the version to be published. XWH participated in the design of the study, has been involved in drafting the manuscript and revising it critically for important intellectual content. All authors read and approved the final manuscript.
